# Maternal Overweight vs. Polycystic Ovary Syndrome: Disentangling Their Impact on Insulin Action in Pregnancy—A Prospective Study

**DOI:** 10.3390/jcm10010035

**Published:** 2020-12-24

**Authors:** Michael Feichtinger, Tina Linder, Ingo Rosicky, Daniel Eppel, Christian Schatten, Wolfgang Eppel, Peter Husslein, Andrea Tura, Christian S. Göbl

**Affiliations:** 1 Wunschbaby Institut Feichtinger, 1130 Vienna, Austria; michael.feichtinger@wunschbaby.at (M.F.); tina.linder@meduniwien.ac.at (T.L.); 2 Division of Obstetrics and Feto-Maternal Medicine, Department of Obstetrics and Gynecology, Medical University of Vienna, 1090 Vienna, Austria; ingo.rosicky@meduniwien.ac.at (I.R.); daniel.eppel@meduniwien.ac.at (D.E.); christian.schatten@meduniwien.ac.at (C.S.); Wolfgang.eppel@meduniwien.ac.at (W.E.); peter.husslein@meduniwien.ac.at (P.H.); 3 Metabolic Unit, CNR Institute of Neuroscience, 35127 Padova, Italy; andrea.tura@cnr.it

**Keywords:** polycystic ovary syndrome, overweight, insulin resistance, pregnancy, glucose homeostasis

## Abstract

Background: To investigate insulin sensitivity and glucose metabolism in pregnant lean and overweight polycystic ovary syndrome (PCOS) patients vs. lean and overweight controls without PCOS. Methods: Prospective cohort study on 67 pregnant women (31 with PCOS and 36 controls, subdivided into overweight or obese and normal weight). All women underwent a 2h-OGTT including glucose, insulin, and C-peptide in early- and mid-gestation and were followed-up until delivery. Results: Insulin sensitivity and glucometabolic parameters were comparable between PCOS patients and controls, whereas marked differences were observed between overweight/obese and lean mothers. Impaired whole-body insulin sensitivity at early pregnancy is mainly a consequence of higher BMI (body mass index; *p <* 0.001) compared to PCOS (*p =* 0.216), whereby no interaction between overweight/obesity and PCOS was observed (*p =* 0.194). Moreover, overweight was significantly associated with gestational diabetes (*p =* 0.0003), whereas there were no differences between women with and without PCOS (*p =* 0.51). Birth weight was inversely related to whole-body insulin sensitivity (rho = −0.33, *p =* 0.014) and positively associated with higher pregestational BMI (rho = 0.33, *p =* 0.012), whereas there was no association with PCOS. Conclusions: Impaired insulin action was mainly a consequence of overweight rather than PCOS. Our data suggest that overweight is more relevant than PCOS for the effects on insulin sensitivity and impaired glucose metabolism.

## 1. Introduction

Polycystic ovarian syndrome (PCOS) is regarded as one of the most common endocrine disorders in women of reproductive age with a wide range of associated adverse conditions. Risk factors for PCOS include overweight, obesity as well as genetic factors [1]. In pregnancy, PCOS was associated with insulin resistance and related pregnancy complications like gestational diabetes mellitus (GDM), macrosomia, and shoulder dystocia [2,3]. In non-pregnant PCOS women, previous studies using the euglycemic-hyperinsulemic clamp showed a higher degree of insulin resistance in lean and obese women compared to the BMI-matched (body mass index) controls [4].

However, due to its close association with increased BMI levels, the role of PCOS as an isolated risk factor for insulin resistance in pregnancy remains controversial. Accordingly, the impact of body weight on PCO-related impairments in glucose disposal and vice-versa is hard to disentangle and published data are conflicted. Even healthy pregnancy is characterized by pronounced changes in maternal insulin secretion and ß-cell function [5]. In particular, maternal overweight and obesity status have been shown to markedly affect glucose metabolism at early gestation, leading to gestational diabetes and adverse pregnancy outcomes [6]. However, pregestational impaired glucose tolerance in PCOS patients has been linked to pregnancy complications independent of their BMI [7,8].

To date, no study has provided a detailed assessment of glucose metabolism in PCOS patients compared to BMI-matched controls in early gestation. Therefore, we aimed to assess differences in insulin sensitivity and further parameters of glucose metabolism in lean and overweight PCOS patients vs. lean and overweight controls without PCOS in early pregnancy. Differences in GDM status and possible associations with birth weight should be additionally assessed.

## 2. Materials and Methods

Sixty-seven healthy pregnant women (31 women with PCOS and 36 controls) were prospectively included between June 2015 and September 2017. PCOS was diagnosed according to the Rotterdam criteria [9]. Exclusion criteria in both groups included acute and chronic illness, preexisting diabetes mellitus, severe anemia, HIV/hepatitis, decreased liver or kidney function, and alcohol abuse or abuse of other toxic substances. Clinical characteristics and summary of laboratory androgen status is provided in the Supplementary Materials (Table S1).

Study participants were further categorized according to their pregestational BMI levels into normal weight (BMIPG < 25 kg/m^2^) and overweight or obese women (BMIPG ≥ 25 kg/m^2^). All participants received a detailed metabolic characterization between 12 + 0 to 22 + 6 weeks of gestation (visit 1) as well as between 24 + 0 to 28 + 6 weeks of gestation (visit 2). At both visits, a 75 g-OGTT (oral glucose tolerance test) was performed including measurements of glucose, insulin, and C-peptide at fasting as well as at 30, 60, 90, and 120 min after ingestion of a 75 g oral glucose load. GDM was diagnosed according to the International Association of the Diabetes and Pregnancy Study Groups recommendations by fasting plasma glucose ≥ 92 mg/dl or 1 h plasma glucose ≥ 180 mg/dL or 2 h plasma glucose ≥ 153 mg/dl [10]. Four patients were diagnosed at V2. Two patients met the IADPSG thresholds already at V1 and were classified as GDM due to elevated self-monitored blood glucose levels during the follow-up examination. Patients with preexisting diabetes were excluded. All laboratory parameters were measured according to the standard laboratory methods at our certified Department of Medical and Chemical Laboratory Diagnostics (http://www.kimcl.at). Plasma glucose levels were measured by the hexokinase method with a coefficient of variation (CV) of 1.3%. The levels of insulin (CV 4–7%) and C-peptide (CV 3–4%) were measured by chemiluminescence immune assays. Calculations of gestational age and sex adjusted percentiles of the Austrian population were based on an analysis of the local fetal growth standard curves. Large for gestational age (LGA) was defined as bodyweight above the 90th percentile. 

### 2.1. Calculation of Parameters of Glucose Homeostasis

Total body insulin sensitivity was assessed by dynamic indices of insulin action from the OGTT data through Matsuda’s composite insulin sensitivity index (ISI-comp) recently developed PREDIM (predicted M) index [11,12]. Thereby, PREDIM provides a prediction of clamp-derived insulin sensitivity (the M value) from the OGTT data. Moreover, the quantitative insulin sensitivity check index (QUICKI) was used to examine insulin sensitivity at fasting state [13]. Insulinogenic indices were used to describe early (ΔInsulin/ΔGlucose 0–30), late (AUCInsulin/AUCGlucose 60–120), and overall insulin response to glucose (AUCInsulin/AUCGlucose 0–120) during the OGTT [14]. The extent to which the pancreatic β-cells can adapt to impaired insulin sensitivity was examined by the product of ISI-comp and AUCInsulin/AUCGlucose 0–120 (sometimes called ISSI-2 or the oral disposition index). In addition, basal insulin secretion (BIS) and total insulin secretion rate (TIS) were assessed by mathematical modeling according to the study by Mari et al. [15].

### 2.2. Statistical Analysis

Categorical variables were summarized by counts and percentages and compared by the Pearson’s Chi-squared test. Continuous scaled variables were summarized by median and interquartile ranges. Rank based inference was used for group-based comparisons due to the skewed distribution of some parameters [16]. Thereby, overweight or obese controls, normal weight PCOS and overweight or obese PCOS women were compared with normal weight control women by using Tukey’s HSD to achieve a 95% coverage probability. The proportional odds cumulative logit model was used as a supportive approach to test the main effects of overweight/obesity vs. normal weight or PCOS vs. controls as well as their interaction (i.e., to test whether the effect of PCOS is modified by BMI levels). Bivariate correlations between ordinal and metric scaled variables were assessed by Spearman’s rank correlation (rho).

Statistical analysis was performed by R (V 3.5.3, R Foundation for Statistical Computing, Vienna, Austria) and contributing packages (especially “nparcomp” and “rms”). The two-sided significance level was set to 0.05.

## 3. Results

A summary of the study sample including glucometabolic parameters at early gestation (visit 1) is provided in Table 1. In the PCOS group, significantly more women conceived after assisted reproduction compared to the control group (26 (83%) vs. 0 (0%) respectively, *p <* 0.001)

**Figure 1:**
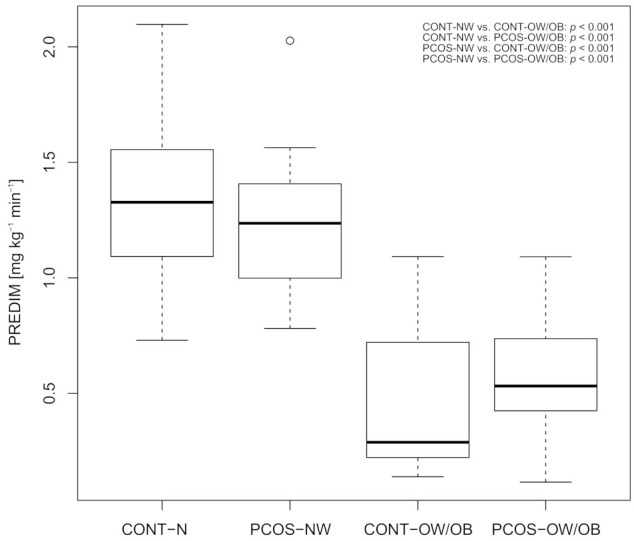
Box-whisker plot for whole body insulin sensitivity assessed by PREDIM. Abbreviations Figure 1: CONT-NW: normal weight controls; CONT-OW/OB: overweight or obese controls; PCOS-NW: normal weight PCOS; PCOS-OW/OB: overweight or obese PCOS; PREDIM: Predicted M.

It was found that impaired insulin action was mainly a consequence of higher body weight: Overweight or obese pregnant women (with and without PCOS) showed markedly lower levels of fasting and dynamic parameters of insulin sensitivity compared to normal weight women (with and without PCOS). However, there were no differences between mothers with PCOS and the controls, regardless of their overweight/obesity status (visualized for whole body insulin sensitivity i.e., PREDIM levels in Figure 1).

The higher amount of insulin resistance observed in overweight or obese mothers at early gestation was accompanied by increased basal and total insulin secretion during the OGTT. Of note, a higher insulin release, particularly at later OGTT periods, was also observed in normal weight mothers with PCOS compared to the healthy controls (*p =* 0.044; Table 1). The proportional odds model was used as a supportive approach and further confirmed our basic conclusions that impaired whole-body insulin sensitivity in early pregnancy (as measured by the PREDIM index) is mainly a consequence of overweight/obesity (*p <* 0.001) as compared to preconceptionally PCOS (*p =* 0.216). Thereby, no effect modification (interaction between overweight/obesity and PCOS status) was observed (*p =* 0.194).

Whole body insulin sensitivity decreased from earlier to later OGTT periods in the total study population (*p =* 0.009). At mid gestation, overweight or obese mothers with PCOS showed significantly lower insulin sensitivity (median PREDIM levels: 0.56, IQR: 0.33–0.67) compared to normal weight PCOS women (1.12, IQR: 1.02–1.20, *p =* 0.002) or controls (1.03, IQR 0.94–1.33, *p =* 0.004), whereas no differences were observed compared to overweight or obese women without PCOS (0.92, IQR 0.42–1.05, *p =* 0.528). Six women developed GDM during the study period (CONT-NW: one patient; PCOS-NW: none; CONT-OW/OB: three; PCOS-OW/OB: four). Overweight or obesity status was significantly associated with the risk of GDM development (*p =* 0.003), whereas diagnosis of PCOS was not (*p =* 0.51). The median weight at the first visit (69.7 kg IQR 62.4–76.5) increased significantly to the second visit (74.0, IQR: 66.0–81.9), however, there were no significant differences between the groups observed.

There were no differences in birth weight percentiles between mothers affected by PCOS vs. mothers without PCOS (median: 61.5, IQR: 26.8–75.5 vs. 54.0, IQR: 18.0–78.0, *p =* 0.416). However, offspring of overweight/obese mothers tended to be significantly larger compared to normal weight mothers (median: 69.0, IQR: 56.5–85.5 vs. 40.5, IQR: 15.8–70.0, *p =* 0.003) as birth weight percentiles were significantly correlated with pregestational BMI (rho = 0.33, *p =* 0.012) and inversely associated with maternal insulin sensitivity (PREDIM: rho = −0.33, *p =* 0.014). Distribution of birth weight percentiles is provided in the Supplementary Materials (Figure S1).

## 4. Discussion

In the present study, glucose metabolism during pregnancy was found to be rather dependent on body weight than on PCOS status. The amount of insulin sensitivity was comparable between pregnant PCOS patients and the controls, whereas marked differences were observed between overweight or obese and normal weight mothers. This resulted in an increased risk for GDM in overweight or obese mothers, which was not observed for PCOS patients.

Due to the strong association of elevated BMI levels with glucose metabolism as well as with PCOS, the impact of PCOS on the parameters of glucose metabolism during pregnancy might be biased. Additionally, the present scientific literature on pregnant PCOS patients is heterogenous with many studies using different definitions of overweight and obesity as possible confounders for glucose deviations during pregnancy. In non-pregnant women, using the euglycemic-hyperinsulemic clamp resulted in a stronger effect of elevated BMI levels (>27 kg/m^2^) than of PCOS in insulin resistance. However, this study also identified impaired insulin action in lean and overweight PCOS patients compared to the BMI-matched controls [4]. In our study, lean PCOS women showed higher insulin secretion during late OGTT, pointing to changed glucose disposal even in lean PCOS patients at early gestation. Of note, in contrast to OGTT results, continuous glucose monitoring data on pregnant PCOS women and controls at the beginning of pregnancy found no differences between PCOS and control pregnant women [17].

In another retrospective study including a relatively large number of lean vs. overweight pregnant PCOS women, none of the lean PCOS women developed GDM in contrast to overweight PCOS women [18]. Our data support these findings since in the present study, the status of PCOS was not associated with the development of GDM, however, the limitation of few GDM cases needs to be considered for the interpretation of these results. Similarly, one recent cohort-study could mainly link GDM to obesity, overweight, and hyperandrogenemia rather to PCOS, further supporting the findings of our study [19]. On the other hand, several large register-based studies could link PCOS to the development of GDM during pregnancy [2,3,20]. However, in those database studies, lean PCOS patients might be underreported, leading to a notable risk of bias.

In PCOS patients developing GDM vs. PCOS patients with normal glucose tolerance during pregnancy, significantly increased insulin and HOMA-IR levels could already be observed preconceptionally and during early pregnancy [21]. Likewise, adverse pregnancy outcomes and gestational diabetes in PCOS patients could be linked to preconceptional impaired insulin sensitivity or impaired glucose tolerance [7,8].

As one retrospective report pointed out, overweight pregnant PCOS patients developed significantly higher fasting insulin levels compared to lean PCOS patients and significantly higher rates of macrosomia could be observed in overweight but not lean PCOS patients [18]. Similarly, one retrospective analysis showed no elevated risk of GDM and elevated birthweight in PCOS patients when corrected for BMI and age [22].

PCO status did not have any impact on birthweight in the present study while we observed that BMI and insulin sensitivity were significantly associated with increased birth weight percentiles.

As a very heterogenous condition, elevated BMI might impact PCOS phenotypes and therefore lead to differences in glucose metabolism. In contrast to overweight PCOS patients, lean PCOS patients present with only mild deviations in their insulin secretion that might be metabolically compensated. With exceeding BMI, patients become more insulin resistant and diabetes may develop when insulin secretion cannot adequately compensate. Accordingly, lean PCOS women seem to have no elevated risk for developing type 2 diabetes or prediabetes compared to the controls [23].

The main strength of our work consists of its prospective character with detailed information of glucometabolic parameters. All patients were recruited in an early phase of their pregnancy and patients received early (12 + 0 to 22 + 6) as well as late (24 + 0 to 28 + 6) OGTT including detailed measures of glucose homeostasis. To the best of our knowledge, this was the first study to prospectively investigate the impact of PCOS on glucose metabolism during pregnancy in overweight and obese and normal weight PCOS patients with such detail. As a weakness of this study, relatively low sample sizes in some subgroups, and especially the low number of GDM cases, have to be acknowledged. Time periods for the first OGTT measurement have been relatively wide (12 − 22 + 6 weeks of gestation), possibly affecting the results. Furthermore, no data on pre-pregnancy glycemic controls could be obtained since recruitment of the patients happened during pregnancy.

## 5. Conclusions

When differentiating between lean and overweight PCOS pregnant women, no significant differences were detected in insulin action at early and late gestation vs. women without PCOS. Higher body weight appears to be more relevant than PCOS for the effects on insulin sensitivity and impaired glucose metabolism.

## Figures and Tables

**Table 1:** Glucometabolic parameters of the study sample at early gestation (visit 1: 12 + 0 to 22 + 6) for normal weight vs. overweight/obese women with polycystic ovary syndrome and controls.

	CONT-NW *n =* 29	PCOS-NW *n =* 21	CONT-OW/OB *n =* 7	PCOS-OW/OB *n =* 10
Age (years)	28.0 (27.0–33.0)	30.0 (29.0–32.0)	33.0 (25.0–37.0)	27.5 (25.5–32.0)
BMIPG (kg/m^2^)	21.8 (20.1–23.4)	22.1 (21.1–23.0)	28.7 (27.2–36.0) *^,†^	29.8 (28.3–34.3) *^,†^
BMIV1 (kg/m^2^)	23.8 (22.0–26.0)	24.0 (22.6–24.6)	32.2 (27.9–37.3) *^,†^	32.2 (29.9–35.8) *^,†^
Parity	0.0 (0.0–1.0)	0.0 (0.0–0.0)	0.0 (0.0–1.0)	0.5 (0.0–1.0)
G0 (mg/dL)	74.0 (72.0–79.0)	74.0 (72.0–78.0)	89.0 (81.0–93.5)	79.0 (76.3–82.8)
G-mean (mg/dL)	95.8 (82.4–106)	100 (84.4–105)	126 (116–140) *^,†^	118 (103–134) *^,†^
I0 (µU/mL)	5.68 (3.11–7.01)	5.94 (4.30–7.78)	16.0 (10.6–19.0) *^,†^	14.6 (11.1–17.4) *^,†^
I-mean (µU/mL)	25.8 (16.7–33.0)	39.3 (26.0–55.5)	81.5 (72.0–97.7) *^,†^	81.0 (46.2–112) *
CP0 (ng/mL)	1.40 (1.20–1.60)	1.50 (1.20–1.70)	2.70 (22.0–2.85) *^,†^	2.55 (2.23–3.20) *^,†^
CP-mean (ng/mL)	4.82 (3.86–5.94)	5.78 (4.38–7.16)	8.12 (7.47–9.92) *^,†^	8.11 (6.04–9.63) *
QUICKI (dimensionless)	0.17 (0.16–0.18)	0.17 (0.16–0.18)	0.14 (0.13–0.15) *^,†^	0.14 (0.14–0.15) *^,†^
ISI-comp (dimensionless)	11.1 (7.20–17.3)	7.71 (5.77–12.2)	2.33 (1.96–3.53) *^,†^	3.29 (2.14–6.36) *^,†^
PREDIM (mg kg^−1^ min^−1^)	1.33 (1.09–1.55)	1.24 (1.00–1.41)	0.29 (0.22–0.72) *^,†^	0.53 (0.43–0.73) *^,†^
Sec-early (µU/mg)	75.2 (32.6–119)	106 (62.6–171)	147 (140–167) *	128 (81.3–232)
Sec-late (µU/mg)	29.3 (16.2–36.6)	46.6 (34.5–66.6) *	75.2 (57.0–84.2) *	71.1 (33.7–104)
Sec-total (µU/mg)	27.7 (20.2–40.0)	42.3 (32.0–47.8)	70.1 (55.6–79.9) *^,†^	65.6 (39.3–92.8) *
BIS (pmol min^−1^ m^−2^)	64.7 (56.7–73.3)	67.4 (54.6–72.4)	116.3 (95.8–118.4) *^,†^	113.6 (102.1–136.2) *^,†^
TIS (nmol/m^−2^)	32.5 (26.5–41.7)	41.9 (30.7–52.2)	55.1 (50.4–65.3) *^,†^	53.8 (42.9–71.1) *
ISSI-2 (dimensionless)	2.59 (2.17–3.59)	3.22 (2.78–3.60)	1.93 (1.33–2.60)	2.00 (1.67–2.51) ^†^

Data are expressed as median and interquartile range. BMIPG, pregestational body mass index; BMIV1, body mass index at the first visit; Values are given for glucose (G), insulin (I), and C-peptide (CP) for fasting as well as the mean values during the OGTT. NW, normal weight; CONT, controls; OW/OB, overweight/obese; QUICKI, quantitative insulin sensitivity check index; ISI-comp, composite index; PREDIM, predicted M; early (sec-early: Δinsulin 0–30 min/Δglucose 0–30 min), late (sec-late: AUC-Insulin/AUC-Glucose (60–120 min)) and overall insulin secretion (sec-total: AUC-Insulin/AUC-Glucose [0–120 min]); BIS, basal insulin secretion rate; TIS, total insulin secretion rate from C-peptide; ISSI-2, oral disposition index.* *p* < 0.05 vs. CONT-NW, † *p* < 0.05 vs. PCOS-NW.

## Data Availability

The data presented in this study are available on request from the corresponding author.

## Supplementary Materials

The following are available online at https://www.mdpi.com/2077-0383/10/1/35/s1, Figure S1: Birth weight percentiles, Table S1: Clinical characteristics and assessment of androgen status.

## References

[1] Norman R.J., Dewailly D., Legro R.S., Hickey T.E. (2007). Polycystic ovary syndrome. Lancet.

[2] Roos N., Kieler H., Sahlin L., Ekman-Ordeberg G., Falconer H., Stephansson O. (2011). Risk of adverse pregnancy outcomes in women with polycystic ovary syndrome: Population based cohort study. BMJ.

[3] Joham A.E., Ranasinha S., Zoungas S., Moran L., Teede H.J. (2014). Gestational diabetes and type 2 diabetes in reproductive-aged women with polycystic ovary syndrome. J. Clin. Endocrinol. Metab..

[4] Stepto N.K., Cassar S., Joham A.E., Hutchison S.K., Harrison C.L., Goldstein R.F., Teede H.J. (2013). Women with polycystic ovary syndrome have intrinsic insulin resistance on euglycaemic-hyperinsulaemic clamp. Hum. Reprod..

[5] Catalano P.M., Tyzbir E.D., Roman N.M., Amini S.B., Sims E.A. (1991). Longitudinal changes in insulin release and insulin resistance in nonobese pregnant women. Am. J. Obstet. Gynecol..

[6] Catalano P.M., Hauguel-De Mouzon S. (2011). Is it time to revisit the Pedersen hypothesis in the face of the obesity epidemic?. Am. J. Obstet. Gynecol..

[7] Wei D., Zhang B., Shi Y., Zhang L., Zhao S., Du Y., Xu L., Legro R.S., Zhang H., Chen Z.J. (2017). Effect of Preconception Impaired Glucose Tolerance on Pregnancy Outcomes in Women With Polycystic Ovary Syndrome. J. Clin. Endocrinol. Metab..

[8] De Wilde M.A., Veltman-Verhulst S.M., Goverde A.J., Lambalk C.B., Laven J.S., Franx A., Koster M.P., Eijkemans M.J., Fauser B.C. (2014). Preconception predictors of gestational diabetes: A multicentre prospective cohort study on the predominant complication of pregnancy in polycystic ovary syndrome. Hum. Reprod..

[9] Rotterdam ESHRE/ASRM—Sponsored PCOS Consensus Workshop Group (2004). Revised 2003 consensus on diagnostic criteria and long-term health risks related to polycystic ovary syndrome (PCOS). Hum. Reprod..

[10] Metzger B.E., Gabbe S.G., Persson B., Buchanan T.A., Catalano P.A., Damm P., Dyer A.R., Leiva A., International Association of Diabetes and Pregnancy Study Groups Consensus Panel (2010). International association of diabetes and pregnancy study groups recommendations on the diagnosis and classification of hyperglycemia in pregnancy. Diabetes Care.

[11] Matsuda M., DeFronzo R.A. (1999). Insulin sensitivity indices obtained from oral glucose tolerance testing: Comparison with the euglycemic insulin clamp. Diabetes Care.

[12] Tura A., Chemello G., Szendroedi J., Gobl C., Faerch K., Vrbikova J., Pacini G., Ferrannini E., Roden M. (2018). Prediction of clamp-derived insulin sensitivity from the oral glucose insulin sensitivity index. Diabetologia.

[13] Katz A., Nambi S.S., Mather K., Baron A.D., Follmann D.A., Sullivan G., Quon M.J. (2000). Quantitative insulin sensitivity check index: A simple, accurate method for assessing insulin sensitivity in humans. J. Clin. Endocrinol. Metab..

[14] Tura A., Kautzky-Willer A., Pacini G. (2006). Insulinogenic indices from insulin and C-peptide: Comparison of beta-cell function from OGTT and IVGTT. Diabetes Res. Clin. Pract..

[15] Mari A., Tura A., Gastaldelli A., Ferrannini E. (2002). Assessing insulin secretion by modeling in multiple-meal tests: Role of potentiation. Diabetes.

[16] Konietschke F., Placzek M., Schaarschmidt F., Hothorn L.A. (2015). nparcomp: An R Software Package for Nonparametric Multiple Comparisons and Simultaneous Confidence Intervals. J. Stat. Softw..

[17] Dmitrovic R., Katcher H.I., Kunselman A.R., Legro R.S. (2011). Continuous glucose monitoring during pregnancy in women with polycystic ovary syndrome. Obstet. Gynecol..

[18] De Frene V., Vansteelandt S., T’Sjoen G., Gerris J., Somers S., Vercruysse L., de Sutter P. (2014). A retrospective study of the pregnancy, delivery and neonatal outcome in overweight versus normal weight women with polycystic ovary syndrome. Hum. Reprod..

[19] West S., Ollila M.M., Franks S., Piltonen T., Jokelainen J., Nevalainen J., Puukka K., Ruokonen A., Jarvelin M.R., Auvinen J. (2020). Overweight, obesity and hyperandrogenemia are associated with gestational diabetes mellitus: A follow-up cohort study. Acta Obstet. Gynecol. Scand..

[20] Mills G., Badeghiesh A., Suarthana E., Baghlaf H., Dahan M.H. (2020). Polycystic ovary syndrome as an independent risk factor for gestational diabetes and hypertensive disorders of pregnancy: A population-based study on 9.1 million pregnancies. Hum. Reprod..

[21] De Wilde M.A., Goverde A.J., Veltman-Verhulst S.M., Eijkemans M.J., Franx A., Fauser B.C., Koster M.P. (2015). Insulin action in women with polycystic ovary syndrome and its relation to gestational diabetes. Hum. Reprod..

[22] Haakova L., Cibula D., Rezabek K., Hill M., Fanta M., Zivny J. (2003). Pregnancy outcome in women with PCOS and in controls matched by age and weight. Hum. Reprod..

[23] Ollila M.M., West S., Keinanen-Kiukaaniemi S., Jokelainen J., Auvinen J., Puukka K., Ruokonen A., Jarvelin M.R., Tapanainen J.S., Franks S. (2017). Overweight and obese but not normal weight women with PCOS are at increased risk of Type 2 diabetes mellitus-a prospective population-based cohort study. Hum. Reprod..

