# Supplementary material for: Maternal Overweight vs. Polycystic Ovary Syndrome: Disentangling Their Impact on Insulin Action in Pregnancy—A Prospective Study
Source: J Clin Med. 2020 Dec 24;10(1):35. doi: 10.3390/jcm10010035 (PMC7795887; doi:10.3390/jcm10010035)
Supplement: Supplementary file 1 [file jcm-10-00035-s001.pdf]

Supplemental Material

Table S1. Clinical characteristics and assessment of androgen status.

	**CONT-NW** *n* = 29	**PCOS-NW** *n* = 21	**CONT-OW** *n* = 7	**PCOS-OW** *n* = 10
PCO-NIH	-	10 (47.6)	-	4 (40.0)
PCO-ROT	-	11 (52.4)	-	6 (60.0)
Tot-Test (ng/ml)	0.53 (0.47–0.76)	0.67 (0.33–1.12)	0.79 (0.37–0.93)	0.92 (0.73–1.92)
Bio-Test (ng/ml)	0.03 (0.02–0.04)	0.03 (0.02–0.06)	0.04 (0.02–0.06)	0.04 (0.04–0.10)*
SHBG (ng/ml)	481 (389–552)	392 (365–446)	338 (318–355)	364 (327–449)
ANST (ng/ml)	4.21 (2.57–5.34)	4.37 (3.40–6.37)	3.61 (2.77–5.47)	4.96 (4.06–5.47)

Data are expressed as median and interquartile range. Four possible phenotypes of PCOS were summarized according to the NIH (PCO-NIH: phenotype A: ovulatory dysfunction + hyperandogenism + polycystic ovaries or phenotype B: ovulatory dysfunction + hyperandrogenism) as well as the Rotterdam criteria, representing two classical and two newer phenotypes (PCOS-ROT; phenotype C: ovulatory dysfunction + polycystic ovary or phenotype D: hyperandrogenism + polycystic ovary). Tot-Test, total testosterone; Bio-Test, bioavailable testosterone; SHBG, sex hormone binding globulin; ANST, androstendione. * $p < 0.05$ vs. CONT-NW; † $p < 0.05$ vs. PCOS-NW; § $p < 0.05$ vs. CONT-OW/OB.

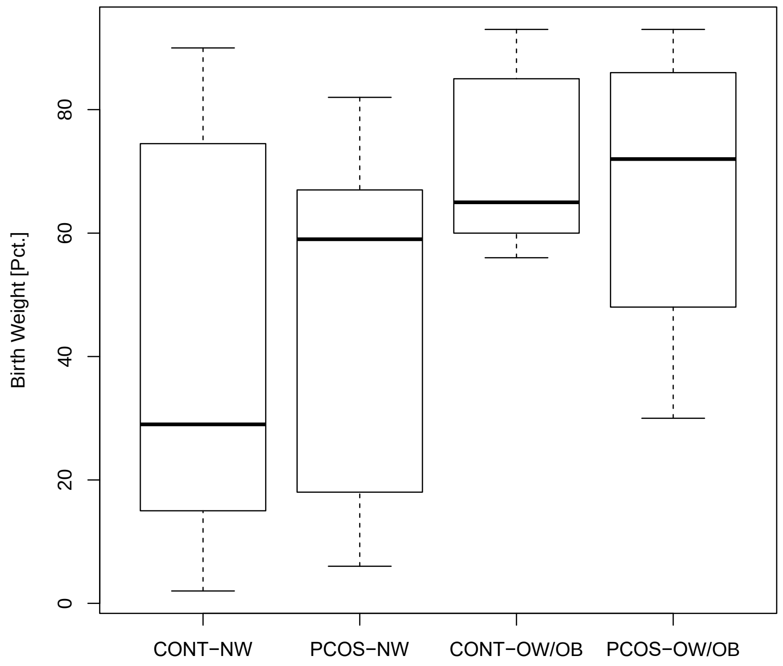

Figure S1. Birth weight percentiles. Abbreviations Figure 1: CONT-NW: normal weight controls; CONT-OW/OB: overweight or obese controls; PCOS-NW: normal weight PCOS; PCOS-OW/OB: overweight or obese PCOS; PREDIM: Predicted M
